# Information-Domain Analysis of Cardiovascular Complexity: Night and Day Modulations of Entropy and the Effects of Hypertension

**DOI:** 10.3390/e21060550

**Published:** 2019-05-31

**Authors:** Paolo Castiglioni, Gianfranco Parati, Andrea Faini

**Affiliations:** 1 IRCCS Fondazione Don Carlo Gnocchi, 20148 Milan, Italy; 2 Istituto Auxologico Italiano, IRCCS, Department of Cardiovascular, Neural and Metabolic Sciences, S.Luca Hospital, 20149 Milan, Italy; 3 Department of Medicine and Surgery, University of Milano-Bicocca, 20126 Milan, Italy

**Keywords:** *SampEn*, cross-*SampEn*, autonomic nervous system, heart rate, blood pressure

## Abstract

Multiscale entropy (MSE) provides information-domain measures of the systems’ complexity. The increasing interest in MSE of the cardiovascular system lies in the possibility of detecting interactions with other regulatory systems, as higher neural networks. However, most of the MSE studies considered the heart-rate (HR) series only and a limited number of scales: actually, an integrated approach investigating HR and blood-pressure (BP) entropies and cross-entropy over the range of scales of traditional spectral analyses is missing. Therefore, we aim to highlight influences of higher brain centers and of the autonomic control on multiscale entropy and cross-entropy of HR and BP over a broad range of scales, by comparing different behavioral states over 24 h and by evaluating the influence of hypertension, which reduces the autonomic control of BP. From 24-h BP recordings in eight normotensive and eight hypertensive participants, we selected subperiods during daytime activities and nighttime sleep. In each subperiod, we derived a series of 16,384 consecutive beats for systolic BP (SBP), diastolic BP (DBP), and pulse interval (PI). We applied a modified MSE method to obtain robust estimates up to time scales of 334 s, covering the traditional frequency bands of spectral analysis, for three embedding dimensions and compared groups (rank-sum test) and conditions (signed-rank test) at each scale. Results demonstrated night-and-day differences at scales associable with modulations in vagal activity, in respiratory mechanics, and in local vascular regulation, and reduced SBP-PI cross-entropy in hypertension, possibly representing a loss of complexity due to an impaired baroreflex sensitivity.

## 1. Introduction

In the 1990s, the approximate entropy (*ApEn*) method paved the way for practically using information-domain techniques in the field of heart rate variability (HRV) analysis [1,2]. Successively its variant, sample entropy (*SampEn*), became particularly popular, providing less biased measures with better relative consistency compared to *ApEn* [3]. *ApEn* and *SampEn* measure the irregularity of a time series by calculating the probability that segments of *m* samples that are similar remain similar when the segment length increases to *m* + 1. This approach was adapted to analyze two series leading to the definitions of Cross-*ApEn* and cross-*SampEn*. Cross-entropy measures may detect interactions between series connected within physiological networks by evaluating their degree of asynchrony [4].

The interest in “information-domain” approaches is because these methods allow the investigation of complexity aspects difficult to evaluate with frequency-domain or time-domain techniques. In fact, the entropy analysis of HRV may offer information on the cardiovascular regulation revealing complexity changes that may reflect an altered integrative autonomic control or modulations from higher brain centers. This information is expected to usefully integrate the traditional methods of HRV in the stratification of the cardiac risk or in monitoring treatments and rehabilitation protocols in the secondary prevention of cardiovascular diseases. Since diseased systems lack adaptability or suffer from an impaired integrative regulation, they are expected to be less “complex” than healthy systems, and therefore, to show lower HRV entropy. However, an intrinsic difficulty in assessing cardiovascular health by entropy is that diseased systems may also show high entropy due to erratic or uncorrelated fluctuations [5]. Therefore, the irregularity of the heart rate, as quantified by *ApEn* or *SampEn*, does not always measure the cardiovascular “complexity”.

In this regard, an important step forward toward a metric of HRV complexity was to separate the temporal scales where entropy components due to erratic fluctuations prevail from those where entropy better reflects the cardiovascular complexity. This was done by introducing the Multi-Scale Entropy (MSE) method, which estimates *SampEn* on progressively coarse-grained series [6]. Successive MSE variants adopted different approaches for low-pass filtering of the heart rate before coarse-graining and for setting the tolerance at each scale [7] or proposed a statistically more robust estimation strategy [8]. Several clinical studies demonstrated the value of MSE and of its variants in assessing HRV alterations in aortic stenosis [7], diabetes [9], atrial fibrillation [10], congestive heart failure [11], long QT syndrome [12], sleep disorders [13], strokes [14,15,16], trauma [17], mental diseases [18], and disorders of consciousness [19]. Animals models of heart failure, hypertension, impaired baroreflex [20], and inflammation [21] also provided evidence of an altered MSE of HRV.

However, an integrated approach for characterizing the MSE of cardiovascular time series is still missing. In fact, very few works also considered the MSE of beat-by-beat blood pressure [9,22,23,24], and none of them investigated the interactions between heart rate and blood pressure in terms of multiscale cross-entropy. Furthermore, all of the HRV studies but one [10] considered a limited number of entropy scales. This is in striking contrast with the frequency range traditionally investigated in HRV studies [25] that includes a very-low-frequency band where the baroreflex control produces its buffering effects on the blood pressure variability [26]. Therefore, our study aims to characterize the cardiovascular regulation with an information-domain approach based on heart-rate and blood-pressure multiscale entropy and cross-entropy, addressing a broad range of scales equivalent to the frequency range of the traditional spectral analyses. To highlight the influences of the circadian modulations on MSE from the higher brain centers, our study will compare different behavioral conditions (nighttime sleep vs. daytime activity); and to point out the role of the autonomic regulation, it will compare normotensive vs. hypertensive groups, in which the autonomic control of blood pressure is known to operate with different efficacy.

## 2. Materials and Methods

### 2.1. Subjects and Data Collection

The study is based on the blood pressure (BP) recordings collected in our previous study on the baroreflex-mediated interactions between heart rate and BP [27]. Briefly, ambulatory intra-arterial BP was measured at the radial site percutaneously inserting a catheter into the radial artery of the non-dominant arm in 8 normotensive participants (NT, 5 males and 3 females, age 43 ± 20 years, referred to our hospital for suspected hypertension, which was excluded after the clinical evaluation) and in 8 subjects with moderate to severe essential hypertension (HT, 7 males and 1 female, age 50 ± 20 years). Clinic systolic BP (SBP) and diastolic BP (DBP) values were 131 ± 6 and 84 ± 4 mmHg (M ± SD) in the NT group, 191 ± 19 and 104 ± 7 mmHg in the HT group.

Recordings started at around 6 pm and lasted 24 h; meal, bed, and recreational times were standardized. In each 24-h recording, we considered two sub-periods each of 5 h duration: The first was selected during daytime in the afternoon, when the subjects were not lying in bed and were free to perform the activities allowed to patients not confined to bed, such as playing cards, watching TV, meeting relatives, or walking on the hospital green (*day* sub-period); the second was selected at night, after 11 PM when the subjects were asleep according to the time schedule of the hospital (*night* sub-period). The recordings were digitized at 12 bits and 170 Hz sampling frequency. Then they were manually edited from movement artifacts, pulse pressure dampening, and premature beats deleting the corresponding portion of the BP tracing. SBP and DBP were calculated beat-by-beat as the highest and lowest BP values in each pulse wave; the pulse interval (PI) was computed as the time interval between consecutive systolic maxima. Missing beats associated with deleted BP signals were not interpolated.

The study protocol was approved by the ethical committee of the Ospedale Maggiore Policlinico di Milano (Milan, Italy) and all the participants gave written informed consent in accordance with the Declaration of Helsinki.

### 2.2. Coarse-Grained MSE and Modified MSE

The MSE method, as originally proposed in [5], estimates entropy at the scale *τ* as *SampEn* of the series coarse-grained with order *τ*. To illustrate the coarse-grained MSE (*cgMSE*) technique and the following modified MSE (*mMSE*) technique, it is useful to summarize the *SampEn* method as revised in [28]. *SampEn* of a time series of *N* samples **X** = {*x*_1_ *x*_2_ *… x_N_*} at the embedding dimension *m* is calculated by constructing the template vectors

(1) $$x_{i}^{m}(\delta )={\left[x_{i},x_{i+\delta },\ldots x_{i+(m-1)\delta }\right]}^{T},\;1\leq i\leq N-m\delta$$

with *δ* the delay between successive components, by calculating the infinity norm distance between all couples of vectors

(2) $$d_{ij}^{m}(\delta )={\left\|x_{i}^{m}(\delta )-x_{j}^{m}(\delta )\right\|}_{\infty },\;1\leq i,\;j\leq N-m\delta ,\;j>i+\delta$$

and by counting the number of matched-pairs, *n_p_*(*m*,*δ*,*r*), i.e., pairs of vectors with a distance lower than a predefined threshold *r*. Then the above steps are repeated for the dimension *m* + 1 obtaining:

(3) $$SampEn(X,N,m,\delta ,r)=-\ln\frac{n_{p}\left(m+1,\delta ,r\right)}{n_{p}\left(m,\delta ,r\right)}$$

see Reference [8]. To calculate *cgMSE* as in [5], coarse-grained series of order *τ* and length *Q* = ⌊*N*/τ⌋, **Y***^τ^* ={ *y*_1_ *y*_2_ *… y_Q_*}, are obtained as:

(4) $$y_{i}=\frac{1}{\tau }\sum_{j=\left(i-1\right)\cdot \tau +1}^{i\cdot \tau }x_{j},\;1\leq i\leq \lfloor N/\tau \rfloor .$$

Setting the tolerance threshold *r* equal to 20% of the standard deviation of **X**, and the distance *δ* = 1 sample, which means that the template vectors are composed by successive samples, *cgMSE* at the scale *τ* is

(5) cgMSE(τ)=SampEn(Yn,⌊Nτ⌋,m,δ=1,r).

Equation (5) indicates that the number of data available for estimating *SampEn* decreases with the scale proportionally to 1/*τ* and the statistical properties of the *cgMSE* estimator quickly deteriorate as the scale increases. Thus, we considered the *mMSE* estimator originally introduced in [8] to estimate MSE on very-short segments of data even if we are dealing with long series, because we aim to investigate a broad range of scales that includes very large *τ*. In the *mMSE* method, the coarse-graining procedure of Equation (4) is replaced with a moving average filter to improve the robustness of the estimate. Accordingly, low-pass filtered series of length *Q = N − τ + 1*, **Z***^τ^* = {*z*_1_ *z*_2_ *… z_Q_*}, are obtained as moving averages of order *τ*:

(6) $$z_{i}=\frac{1}{\tau }\sum_{j=i}^{i+\tau -1}x_{j},\;1\leq i\leq N-\tau +1$$

and the *mMSE* at scale *τ* is the *SampEn* of **Z***τ* with delay *δ* = *τ*:

(7) $$mMSE(\tau )=SampEn\left(Z^{\tau },N-\tau +1,m,\delta =\tau ,r\right).$$

At any order *τ* > 1 the number of data for estimating *SampEn* is greater with *mMSE* than with *cgMSE*. To verify that *mMSE* provides statistically more robust estimates, we generated a series of white Gaussian noise, 1/f (or pink) noise, and Brownian motion (or brown noise). For each type of noise, we generated series of length 1000, 10,000, and 100,000 samples, and calculated *mMSE* and *cgMSE* for scales *τ* ≤ 40.

**Figure 1:**
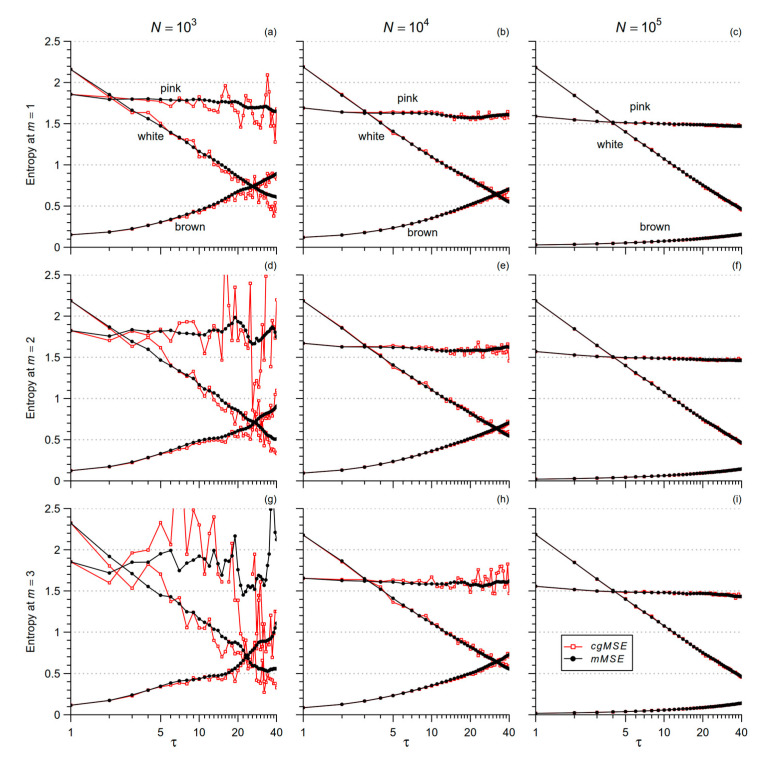
Comparison of Multiscale Entropy estimators for white, pink, and brown noise. Time series of different length *N* were analyzed with the *cgMSE* (red lines) and *mMSE* (black lines) algorithms for 3 embedding dimensions *m*. Panels (**a**,**d**,**g**): *N =* 10^3^; panels (**b**,**e**,**h**): *N =* 10^4^; panels (**c**,**f**,**i**): *N =* 10^5^; panels (**a–c**): *m* = 1; panels (**d–f**): *m* = 2; panels (**g–i**): *m* = 3. Note the more stable estimates with longer *N*, lower *m*, and with the *mMSE* algorithm, being *cgMSE* unable to provide estimates for pink noise at the larger scales when *N* = 10^3^. At all the scales, independently from the algorithm, the estimates decrease with *N* for pink and brown noise.

**Figure 2:**
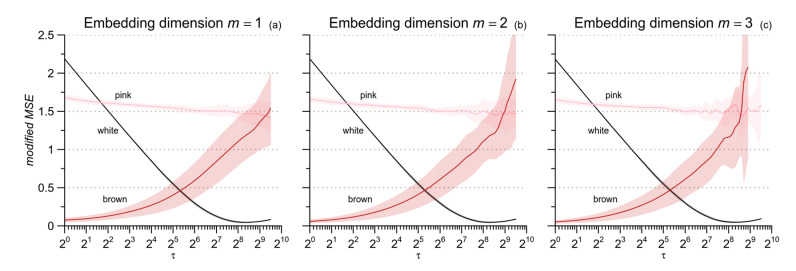
Modified Multiscale Entropy for white, pink, and brown noises. Mean value ± SD for ten series of *N* = 2^14^ samples and scales *τ* ≤ 724 samples; (**a**): *m* = 1; (**b**): *m* = 2; (**c**): *m* = 3. For these three noise processes, the estimation variability is greater at the larger scales and increases with *m*, while the expected value of the estimates does not depend on *m*.

Figure 1 shows that the uncertainty of the estimate is greater for *cgMSE* (which was unable to estimate the larger scales for *N* = 1000) while the expected values are similar for the two estimators. This suggests to replace coarse-grained series with low-pass filtered and delayed series, as in [8]. Figure 1 also highlights that at any given scale *τ*, the entropy estimate decreases as *N* increases for the pink and brown noises, but not for the white noise. Pink and brown noises, unlike the white noise, are non-stationary fractional Brownian motions whose standard deviation increases with the length of the series (see Figure 6 in [29]). Since *r* is a fraction of the standard deviation, the tolerance increases with *N* for fractional Brownian motions while it does not depend on *N* for stationary processes. Therefore, to avoid the confounding factor of the series length, we decided to compare conditions and groups considering beat-by-beat series of the same length, which was set to 2^14^ (=16,384) beats. To empirically evaluate the range of scales estimable by *mMSE* with series of this length, we synthesized 10 series of white noise, 10 series of pink noise and 10 series of brown noise, for each of 16,384 samples; and we calculated mean and standard deviation of *mMSE(τ)* up to *τ* = 724 and 1 ≤ *m* ≤ 3 (to reduce the computation load, we calculated *mMSE* for all *τ* when *τ* ≤ 16 and for *τ* exponentially distributed over the scale axis when *τ* > 16, with a density of 8 estimates at each doubling of the axis). Figure 2 suggests that the length of 16,384 beats allows estimating scales up to around *τ* = 600 beats, with the exception of *m* = 3 where *mMSE* of brown noise appears limited to 512 samples.

### 2.3. MSE of Cardiovascular Series: From Scales in Beats to Temporal Scales in Seconds

**Figure 3:**
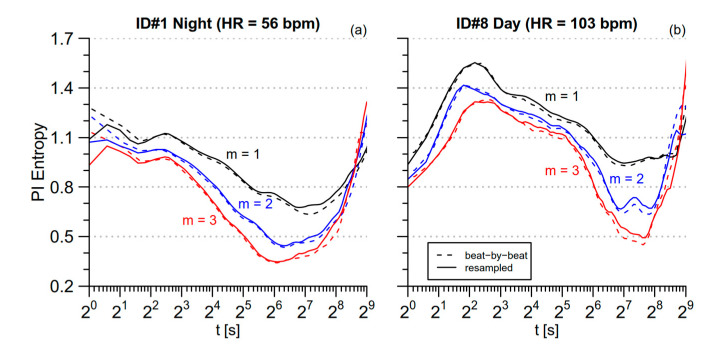
Modified Multiscale entropy for the original and the evenly oversampled beat-by-beat series. Estimates are shown for segments of 2^14^ s and for three embedding dimensions. Estimates on the beat-by-beat series (dashed lines) are plotted vs. the scale *t*, in seconds, calculated by multiplying *τ* in beats by the mean PI, in seconds; estimates after interpolation and oversampling at 2 Hz (continuous lines) are plotted vs. the scale *t*, in seconds, calculated dividing *τ*, in number of samples, by the sampling frequency, in Hz; (**a**) the most bradycardic segment, during nighttime sleep; (**b**) the most tachycardic segment, during daytime activities.

Since the PI, SBP, and DBP series are sampled on a beat-by-beat basis, the MSE algorithms provide estimates on scales *τ* with units in number of beats and not in seconds. This means that when groups or conditions with different heart rates are compared, the same scale in beats corresponds to different scales in seconds; and that the range of scales associated with a given frequency band of the traditional HRV spectral analysis depends on the mean heart rate. To overcome these problems, we mapped the scales from beats to seconds with the transformation:

(8) t=τ×〈PI〉,

where <PI> is the mean PI, in seconds, over the series. However, a possible problem not addressed by Equation (8) is the distortion due to considering the sampling rate constant when actually the beat-by-beat sampling is intrinsically uneven. This issue was considered in [10], which evaluated *cgMSE* after interpolating and oversampling the beat-by-beat series at 2 Hz. To quantify the distortion due to the uneven sampling, we compared two *mMSE*(*t*) estimates: The first was obtained by applying the *mMSE* method on the original beat-by-beat series and by mapping the scale axis from the beat-scale to the time-scale according to Equation (8); the second estimate was obtained by applying the *mMSE* method on a spline-interpolated, evenly-resampled series at 2 Hz. Figure 3 compares the estimates of PI *mMSE* without and with evenly oversampling for the two series of 16,384 s with the lowest and highest mean heart rate among those selected for our analysis: subject 1 during nighttime sleep (the most bradycardic series) and subject 8 during daytime activities (the most tachycardic series). Discrepancies between the two approaches are negligible almost at all the scales in both the conditions. This is likely due to the selection of data segments that avoided periods with substantial long-term trends of the mean heart rate, such as during the transition from wake activities to sleep (for this reason we might have found greater distortions if we had analyzed the whole 24-h period). However, Figure 3a also shows that estimates after interpolation and oversampling are substantially lower at *t* < 3 s for the bradycardic subject; this is probably due to the correlation among samples introduced by the spline interpolation. Therefore, we decided not to interpolate and oversample evenly the beat-by-beat series to avoid possible distortions at the shorter scales, considering acceptable the discrepancies between unevenly- and evenly-sampled series at least for the *day* and *night* data segments selected in our study. Furthermore, since the series are not oversampled evenly, the *mMSE* at the scale *τ* = 1 beat directly corresponds to *SampEn*.

### 2.4. Low-Pass Filtering for mMSE Estimates

The coarse-graining procedure in *cgMSE* removes components with scales shorter than *τ* beats by a moving average over *τ* samples (see Equation (4)). The moving average filter has a poor frequency response due to a wide transition band, and it has been suggested to replace it with a zero-phase, 6-th order low-pass Butterworth filter with cut-off frequency *f_c_* = 0.5/*τ* [7]. This suggestion, originally proposed for *cgMSE*, is also valid for *mMSE* that employs a moving average filter in Equation (6). To quantify the effect of the narrower transition band of the Butterworth filter on *mMSE*, we replaced the **Z***^τ^* series of Equation (6) with **B***^τ^*, the output of the filter proposed in [7] applied on the original **X** series. However, we found a numerical instability calculating the filter coefficients for scales *τ* ≥ 380 beats (by contrast, the moving average filter was always stable with coefficients equal to 1/*τ*). To overcome this problem, we used a two-step procedure when *τ* ≥ 380 beats. In the first step, the **X** series was low-pass filtered with *f_c_* = 0.5/4 and downsampled by a factor of 2. Since the scale *τ* of the original series of length *N* = 2^14^ samples corresponds to the scale *p* = *τ*/2 on the downsampled series of length 2^13^ samples, we could design the Butterworth filter with *f_c_* = 0.5/*p*. In the second step, the downsampled series was low-pass filtered, spline-interpolated and oversampled by a factor of 2 to reach the original length of 2^14^ samples again. Finally, the *mMSE* was calculated as:

(9) $$mMSE(\tau )=SampEn\left(B^{\tau },N,m,\delta =\tau ,r\right)$$

with **B**^1^ = **X**.

**Figure 4:**
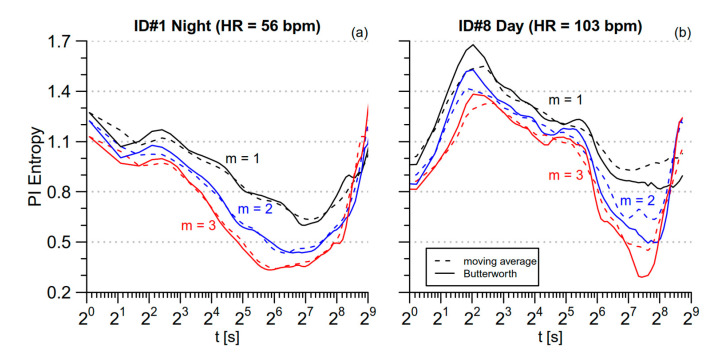
Comparison between moving average and Butterworth filter in estimating the modified MSE. The same beat-by-beat PI series of Figure 3 are considered; (**a**) the most bradycardic segment, during nighttime sleep; (**b**) the most tachycardic segment, during daytime activities.

Figure 4 compares *mMSE* obtained with the moving average and the Butterworth filters for the same data segments of Figure 3. The Butterworth filter provides estimates with better temporal resolution thanks to its narrower transition band. In particular, Figure 4 points out that it better locates the maximum around *t* = 4 s for both the segments as well as the maximum and the minimum at *t* = 40 s and *t* = 180 s, respectively, for the tachycardic segment.

**Figure 5:**
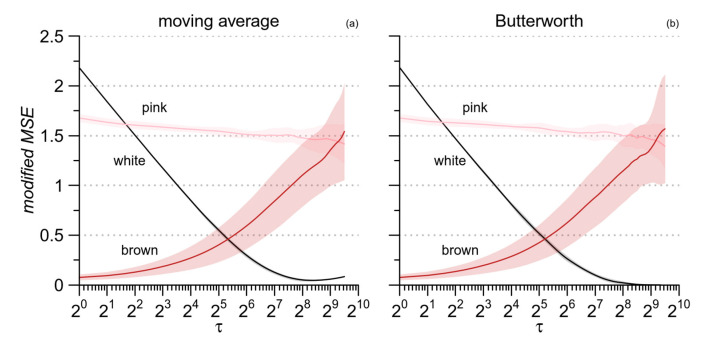
Comparison between moving average and Butterworth filter in estimating the modified MSE with *m* = 1 for the same noise processes of Figure 2. (**a**) moving average filter; (**b**) Butterworth filter.

The better performance of the Butterworth filter appears clearly in Figure 5 also, which plots the *mMSE* estimates for the synthesized noises of Figure 2 as obtained by the two filters. The major discrepancy regards the entropy of white noise, expected to decrease monotonically with *τ* up to a minimum close to zero at the largest scale. This pattern appears clearly with the Butterworth filter (Figure 5b). By contrast, when the moving average is used, the lowest entropy, slightly greater than zero, is reached at scales around 2^8^, and at the largest scales, the entropy estimate even tends to increase (Figure 5a). This is likely due to a residual high-frequency variability not properly removed by the moving-average filter. Therefore, we applied the *mMSE* method replacing the moving average with the Butterworth filter.

To summarize, our MSE method differs from multiscale estimators of previous studies because (1) it considers temporal scales, in seconds, rather than scales in number of beats; (2) it is based on the modified MSE approach [8] but replacing the moving average with a Butterworth filter. Unlike the refined-MSE [7], which originally proposed the Butterworth filter for coarse-graining, our MSE estimator does not downsample the series and does not update the tolerance threshold at each scale by re-calculating *r* as a fraction of the standard deviation of the filtered series. This latter choice is based on our previous analysis suggesting that a fixed threshold may provide more stable estimates and better highlight physiological differences between heart-rate and BP dynamics [23].

### 2.5. Multiscale Cross-Entropy between SBP and PI

We estimated the multiscale cross-*SampEn* between PI and SBP similarly extending the cross-*SampEn* estimator [3] to multiple scales. Before cross-entropy analysis, the mean was removed and each series normalized to unit variance dividing each value by the standard deviation of the series. Called **P** = {*p*_1_ *p*_2_ *… p_N_*} and **S** = {*s*_1_ *s*_2_ *… s_N_*} for the normalized PI and SBP series of *N* beats, we calculated the template vectors for a given embedding dimension *m*

(10) $$\begin{array}{c} p_{i}^{m}(\delta )={\left[p_{i},p_{i+\delta },\ldots p_{i+(m-1)\delta }\right]}^{T}\\ s_{i}^{m}(\delta )={\left[s_{i},s_{i+\delta },\ldots s_{i+(m-1)\delta }\right]}^{T} \end{array},\;1\leq i\leq N-m\delta$$

the distances between all couples of vectors

(11) $$d_{ij}^{m}(\delta )={\left\|p_{i}^{m}(\delta )-s_{j}^{m}(\delta )\right\|}_{\infty },\;1\leq i,\;j\leq N-m\delta$$

and the number of pairs of vectors with distance lower than a threshold *r*, *n_p_*(*m*,*δ*,*r*). We calculated the same quantities for *m* + 1 defining cross-*SampEn* as

(12) $$XSampEn(P,S,N,m,\delta ,r)=-\ln\frac{n_{p}\left(m+1,\delta ,r\right)}{n_{p}\left(m,\delta ,r\right)}$$

Unlike cross-*ApEn*, cross-*SampEn* is direction independent, which means that *XSampEn*(**P**,**S**,*N*,*m*,δ,*r*) = *XSampEn*(**S**,**P**,*N*,*m*,δ,*r*) [3]. To evaluate the cross-*SampEn* at the scales *n*, we set *r* = 0.20 and low-pass filtered **P** and **S** using the Butterworth filter employed for *mMSE* with low-pass frequency *f_c_* = 0.5/*τ*, obtaining the filtered series **P***^τ^* and **S***^τ^* (where **P**^1^ = **P** and **S**^1^ = **S**). The modified multiscale cross entropy, *mMXSE*, at the scale *τ ≥* 1 was calculated as:

(13) $$mMXSE\left(\tau \right)=XSampEn(P^{\tau },S^{\tau },N,m,\delta =\tau ,r)$$

At *τ* = 1, *mMXSE* coincides with cross-*SampEn*. The scales were mapped from beats *τ* into times *t*, in seconds, according to Equation (8).

### 2.6. Statistical Analysis

*SampEn* and cross-*SampEn* estimates were compared between NT and HT groups and between *day* and *night* conditions by a repeated-measures linear mixed-effects model, applied on ranks to handle possible violations of the hypothesis of normality of the residuals. Preliminary to the statistical assessment of multiscale entropies, the *mMSE*(*t*) and *mMXSE*(*t*) functions were interpolated and resampled to obtain 50 estimates at scales *t* exponentially distributed over the temporal axis between 2 s and 512 s. To identify the temporal scales where differences between the *day* and *night* conditions may occur, we calculated the Wilcoxon signed rank test between conditions at each *t*; this was done separately for the NT and the HT groups. The test is based on the statistics of the quantity *W*, defined as the sum of the ranks of differences between pairs, taken with their sign [30]. Then, to identify the scales where differences between the NT and the HT groups may occur, we calculated the statistics of the Wilcoxon rank-sum test between groups at each *t*; this was done separately for the *night* and *day* conditions. The test is based on the statistics of the quantity *V*, defined as the lowest sum of the ranks between two groups [30]. The 5% value of the *W* and *V* statistical distributions was taken as the statistical significance threshold to reject the null hypothesis for the comparison between two conditions in each group, or between two groups in each condition; the threshold was adjusted by the Bonferroni correction when considering multiple comparisons.

Both these tests do not require the assumption of normal distributions. Tests were performed with “R: A Language and Environment for Statistical Computing” software package (R Core Team, R Foundation for Statistical Computing, Vienna, Austria, 2017).

## 3. Results

### 3.1. PI Entropy

**Figure 6:**
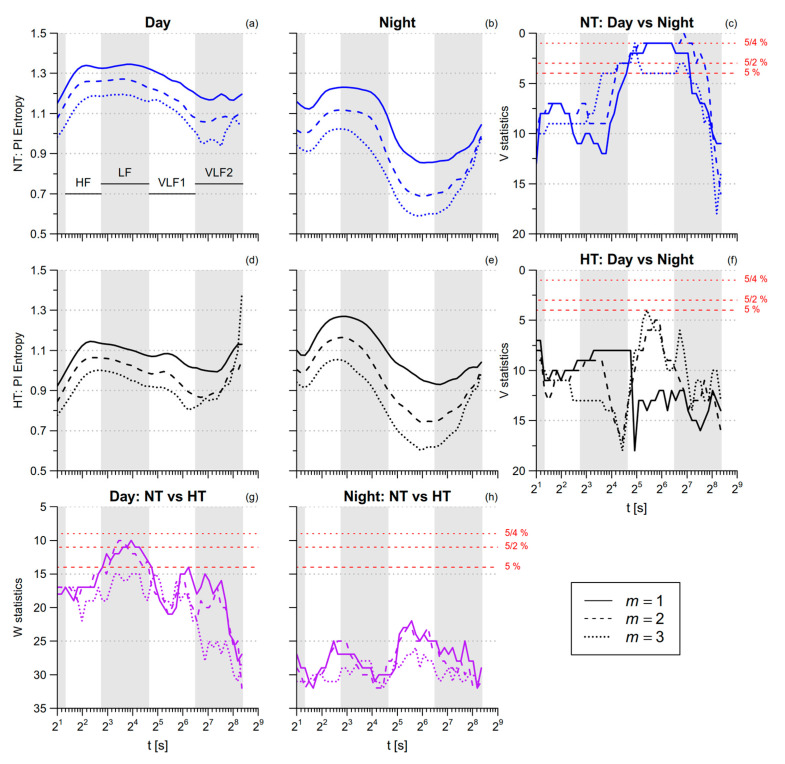
Multiscale Sample Entropy of PI in normotensive (NT) and hypertensive (HT) groups, during day and night conditions. Average modified multiscale entropy *mMSE*(*t*) over eight NT and eight HT participants during nighttime sleep (panels (**b**,**e**)) or daytime activities (panels (**a**,**d**)), for embedding dimensions *m* between one and three; as a reference, gray bands in each panel show the ranges of scales corresponding to the high-frequency (HF), low-frequency (LF), and very-low-frequency (VLF) bands of traditional spectral analysis (with VLF = VLF1 + VLF2, see text). Panels (**c**,**f**): Wilcoxon signed-rank statistics *V* for the comparison between conditions, separately in NT and HT groups; panels (**g**,**h**): Wilcoxon rank–sum statistics *W* for the comparison between groups, separately in day and night conditions. The lower red horizontal line is the 5th percentile of the *V* or *W* distributions: when the distribution is above this threshold, the difference is statistically significant at *p* < 5% and the hypothesis of similar entropies for a given condition and a given group is rejected; the intermediate red horizontal line corresponds to the same significance threshold after Bonferroni correction for two comparisons (NT vs. HT for both conditions, day vs. night for both groups); the upper red line corresponds to the Bonferroni correction of the statistical threshold for all the four comparisons simultaneously.

Table 1 reports that PI *SampEn* is greater during nighttime than in daytime. Figure 6 shows the profiles of PI multiscale entropy for *t* between 2 s and 334 s. This range includes the scales associated with the traditional high-frequency (HF, 2.5 ≤ *t* < 6.7 s), low-frequency (LF, 6.7 ≤ *t*< 25 s), and very-low-frequency (VLF, 25 ≤ *t* < 333.3 s) bands of the HRV spectral analysis. As a reference, the position of these bands is displayed in each panel. As suggested in [10], the VLF band is split into a VLF1 (25 ≤ *t* < 90 s) and a VLF2 (90 ≤ *t* < 333.3 s) sub-bands. At scales up to the LF range, PI multiscale entropy does not differ significantly between *day* and *night*. By contrast, in the VLF1 band PI multiscale entropy is significantly lower at *night*, but in the normotensive group only. During daytime activities, the PI entropy tends to be lower in the hypertensive participants, the differences being more significant between 7 and 24 s, a range of scales that falls within the LF band.

### 3.2. Blood Pressure Entropy

**Figure 7:**
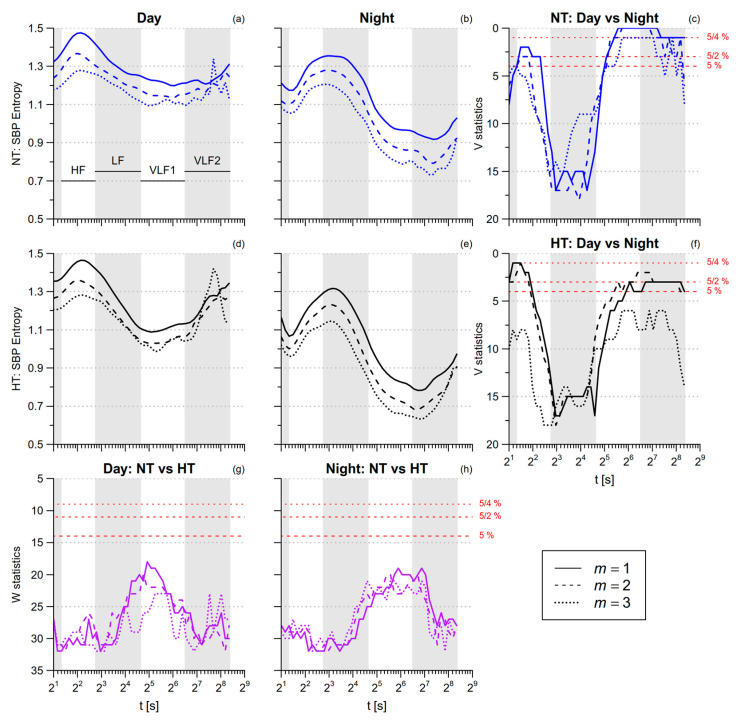
Multiscale Sample Entropy of SBP in normotensive (NT) and hypertensive (HT) groups, during *day* and *night* conditions. Panels (**a**,**b**,**d**,**e**): average *mMSE*(*t*) by groups and conditions for 1 ≤ *m* ≤ 3. Panels (**c**,**f**): signed-rank statistics *V* for the comparison between conditions; Panels (**g**,**h**): rank-sum statistics *W* for the comparison between groups. See also Figure 6.

**Figure 8:**
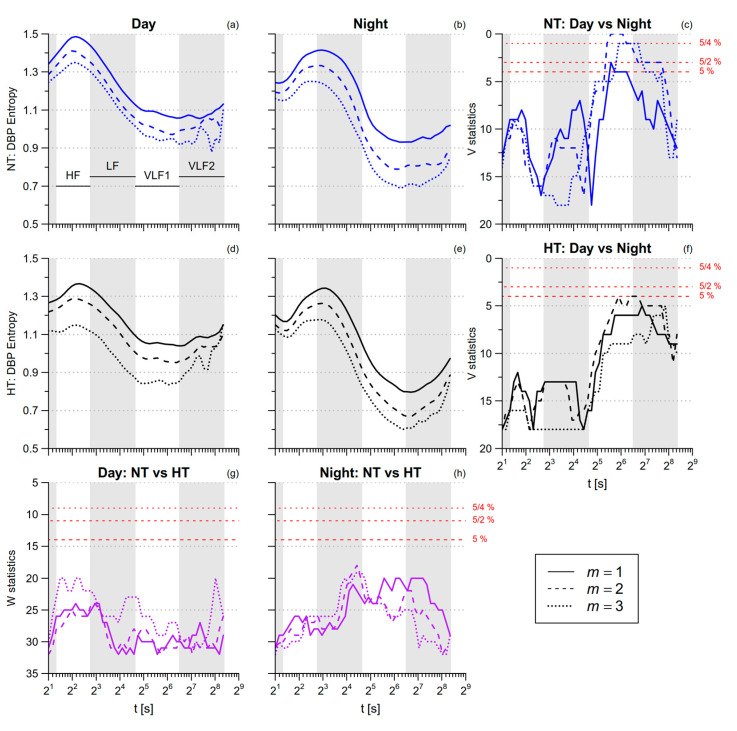
Multiscale Sample Entropy of diastolic blood-pressure (DBP) in normotensive (NT) and hypertensive (HT) groups, during *day* and *night* conditions. Panels (**a**,**b**,**d**,**e**): average *mMSE*(*t*) by groups and conditions for 1 ≤ *m* ≤ 3. Panels (**c**,**f**): signed–rank statistics *V* for the comparison between conditions; Panels (**g**,**h**): rank-sum statistics *W* for the comparison between groups. See also Figure 6.

SBP *SampEn* is greater during nighttime sleep than during daytime activities (Table 1). By contrast, SBP multiscale entropy is lower during nighttime sleep in the HF and VLF ranges (Figure 7). DBP *SampEn* and DBP multiscale entropy in the HF range do not differ between *day* and *night*, while in the VLF range DBP multiscale entropy tends to be lower at night (Figure 8). The comparison between NT and HT groups does not reveal significant differences, neither for SBP nor for DBP.

### 3.3. SBP-PI Cross-Entropy

**Figure 9:**
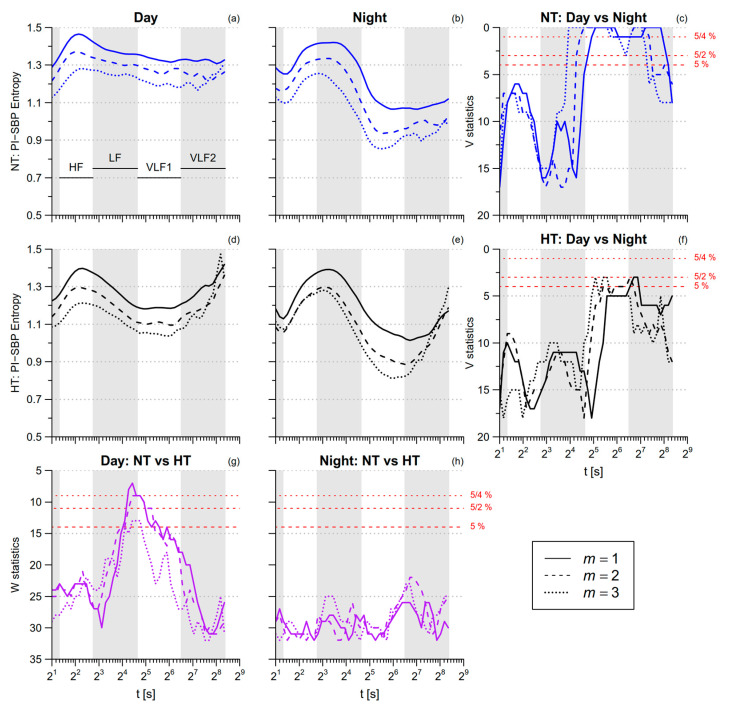
Multiscale Cross Sample Entropy between PI and SBP during *day* and *night* conditions. Panels (**a**,**b**,**d**,**e**): average modified multiscale cross entropy (*mMXSE*(*t*)) by groups and conditions for 1 ≤ *m* ≤ 3. Panels (**c**,**f**): signed-rank statistics *V* for the comparison between conditions; panels (**g**,**h**): rank–sum statistics *W* for the comparison between groups. See also Figure 6.

The SBP-PI cross-*SampEn* is greater during nighttime sleep than during daytime activities (Table 1). Significant *night*/*day* differences in multiscale cross-entropy (Figure 9) regard the VLF scales in the normotensive group, with lower entropy at *night*. The *night*–*day* differences are less pronounced in the hypertensive group because of their lower cross-entropy during daytime activities, the difference between the NT and HT groups being more significant at scales from 15 s to 45 s, i.e., over the LF and VLF1 ranges.

Table 2 summarizes the results showing the average values of multiscale PI, SBP and DBP entropy, and SBP-PI cross entropy, over the scales corresponding to the HF, LF, VLF1 and VLF2 bands, separately by groups and conditions.

## 4. Discussion

Our work presents the first joint analysis of heart rate and blood pressure multiscale entropy, designed to address the same range of scales of the traditional power spectral methods. By considering 24-h recordings in normotensive and in hypertensive participants, our information-domain analysis (1) provided us with a more detailed description of night-and-day modulations of cardiovascular entropy than achievable with mono-scale *SampEn* and cross-*SampEn*, and (2) allowed us to report a loss of cardiovascular complexity in hypertension. In particular, the following results deserve to be discussed.

### 4.1. Day-Night Modulations in Normotensive Subjects

*SampEn*. The comparison between *day* and *night* conditions reveals that the circadian modulations of entropy depend strongly on the scale. The fastest scales are described by *SampEn*, which coincides with the multi-scale entropy at the scale of one beat. As to PI, *SampEn* is much greater during nighttime sleep than during daytime activities (Table 1). This result can be explained by the positive association between the heart-rate entropy and the vagal tone or by the inverse association with the sympatho/vagal balance. This was demonstrated by a greater heart-rate permutation entropy in supine than in orthostatic position [32], being the vagal tone higher in supine than in standing posture; and by a lower heart-rate *SampEn* after pharmacological vagal blockade [33]. The vagal tone is expected to be greater sleeping at night than during daytime activities (1) because of the lying posture and (2) because of the cardiac sympatho/vagal balance activations during the daily activities, and this would explain the greater PI *SampEn* at *night.* The vagal tone does not influence the DBP dynamics, which mainly depend on the modulations of peripheral resistances, and coherently no differences between *day* and *night* are found for DBP *SampEn*. The tendency of SBP *SampEn* to increase during nighttime sleep might reflect the greater PI *SampEn* through beat-by-beat modulations of stroke volume.

*HF range*. At slightly greater scales than one beat, i.e., in the HF range, *day-night* modulations of multiscale entropy differ markedly from those of *SampEn*. In fact, unlike the PI *SampEn*, which is greater in *night* with high statistical significance, the PI multiscale entropy does not differ between *day* and *night* in the HF range (Figure 6c). Furthermore, both *SampEn* and HF entropy of SBP differ significantly between *day* and *night* (Figure 7c), but while SBP *SampEn* is greater in *night*, the HF multiscale entropy of SBP is lower in *night*. The profiles of multiscale entropy vs. *t* explain these discrepancies: During *day*, the multiscale entropy increases with *t* reaching a maximum in the HF range while, during *night*, it decreases with *t* reaching a minimum in the HF range. The relative minimum of SBP entropy in the HF range during *night* could be due to the respiratory fluctuations: in fact, respiratory driven oscillations of SBP fall into the HF band and are more regular during sleep than during daytime activities. As to DBP entropy, *night*–*day* differences at the shorter scales are not significant (Figure 8c), coherently with the lower amplitude of the respiratory oscillation in DBP than in SBP [34]. In addition, the relative minimum of PI entropy in the HF range could be explained by a more regular respiratory sinus arrhythmia during sleep.

*VLF1 range*. The more evident differences between *day* and *night* are found around the VLF1 range, encompassing scales between half a minute and a few minutes. These differences similarly regard heart-rate and BP multiscale entropy as well as the SBP-PI multiscale cross-entropy and consist of a substantial reduction of entropy during nighttime sleep. The VLF1 range may be linked to the relatively slow mechanisms of vascular regulation that control the local blood flow in individual districts. We may expect that the regulation of local blood flows from the higher brain centers is less engaged while sleeping, in the lying position, than during the afternoon activities performed freely moving within the hospital. Both changes in BP or in heart rate may produce changes in blood flow. This would explain the lower VLF1 entropy of both BP and PI during nighttime sleep as due to the more constant supply of blood flow to the individual vascular districts provided by the mechanisms of integrative cardiovascular regulation which is, consequently, reflected in a more regular dynamics of BP and PI.

### 4.2. Hypertension and Entropy

*MSE.* Our study provides evidence of a loss of heart rate complexity in hypertension. In fact, the multiscale entropy of PI is lower in the HT group during the daytime activities (Figure 6a,d), the difference reaching the statistical significance at scales around the LF range (Figure 6g). By contrast, the multiscale entropy of the NT and HT groups are very similar during nighttime sleep. In this regard, a previous study showed that changing posture from supine to 90° head-up tilt increased the sympatho/vagal balance less in hypertensive than in normotensive patients, so that the hypertensive group, compared to normotensive controls, had a lower LF normalized power in the upright posture but not in the supine posture [35]. This finding was explained by the lower baroreflex sensitivity in the hypertensive participants, which caused a lower sympathetic activation in response to the upright position [35]. We recently showed that our HT group, compared to the NT one, also has a lower baroreflex sensitivity [27]: thus, we could similarly hypothesize that the lower LF entropy in the HT group is a consequence of the impaired baroreflex sensitivity that caused a lower autonomic activation in response to the afternoon activities. Because the PI multiscale entropy is lower in the HT group during *day* but not during *night,* the *day–night* differences, significant in the NT group, do not reach the statistical significance in the HT group (Figure 6f).

*XMSE*. However, the more pronounced alterations associated with hypertension regard the SBP-PI cross-entropy during daytime activities, particularly at scales in-between the LF and the VLF1 bands (Figure 9g). Cross-*SampEn* measures the degree of asynchronicity between two time series (the lower is the cross-*SampEn*, the more synchronized are the two series). Accordingly, not only are the PI fluctuations more regular in the HT group because of their lower entropy but they are also better synchronized with the SBP fluctuations. We recently showed in the same participants that the feedback baroreflex coupling of PI with SBP is reduced in the HT group while the feedforward mechanical coupling of SBP with PI is preserved [27]. Since cross-*SampEn* is not direction dependent, it does not distinguish the contribution of the feedforward PI-to-SBP coupling from the contribution of the feedback SBP-to-PI coupling. The two contributions are responsible for rather different patterns of coupling: In fact, an increase/decrease of PI is associated with a decrease/increase of SBP due to the feedforward coupling, while it is associated with the opposite pattern, i.e., an increase/decreases of SBP, due to the feedback coupling. Thus, we may hypothesize that the superposition of two contributions with such different dynamics increases the asynchronicity rather than the synchronization of the two signals. This would suggest that the greater PI-SBP synchronization in hypertensive individuals reflects the prevailing mechanical coupling between PI and SBP. The lower PI-SBP entropy during daytime activities at scales between 15 s and 45 s would thus reflect a loss of cardiovascular complexity in hypertension.

### 4.3. Limitations and Conclusions

Technical and methodological aspects should be considered regarding the reproducibility of our entropy measures. We recorded BP invasively at the radial artery: If the less accurate but more practical noninvasive instrumentation for measuring continuous BP at the finger site is used, SBP entropy might differ around the LF range because of the amplification of the LF fluctuations of SBP at the digital artery [36]. Moreover, if the beat duration is measured as the R-R interval from the electrocardiogram, differences with the PI entropy might be observed at the shorter scales because of the difference between the PI and the R-R interval spectral powers in the HF band [37]. Another methodological aspect regards the use of the classic “fixed-threshold” approach, as originally proposed for the multiscale entropy [6], which means that the entropy is calculated with the same tolerance threshold *r* at all the scales, as in Equation (5). Other authors proposed using a tolerance threshold that varies at each scale as the standard deviation of the coarse-grained series [7]: With such a “varying-threshold” approach, the results could be different [23]. Finally, we selected two groups of NT and HT subjects of the same size with non-dissimilar age range (*p* = 0.41 after Student’s *t*-test) and sex composition (*p* = 0.57 after Fisher’s exact test); however, with a larger population of participants, we could have excluded more safely possible age and gender biases in our results.

In conclusion, we demonstrated the feasibility of an information-domain evaluation of the beat-by-beat BP dynamics encompassing the same scales of the traditional spectral analysis from recordings of a few hours duration. Furthermore, our multiscale analysis on sub-periods selected within the 24 h demonstrated night-and-day differences in the structure of the cardiovascular entropy. Considering the scales in which the circadian differences were observed, they could be separately associated with distinct physiological mechanisms, which likely are the vagal activity for the fastest scales of PI entropy, the respiratory mechanics for the HF scales of SBP entropy, and the local blood flow regulation for the VLF1 scales of heart rate and BP. From a clinical perspective, of note is the substantial reduction of the PI-SBP cross-entropy in the hypertensive group. Since the reduction was detected at scales shorter than 45 s and was elicited by daily activities, our result suggests the possibility that the SBP-PI cross-entropy measured at these scales during an autonomic challenge may help to quantify alterations in the cardiovascular control mechanisms. Thus, future autonomic tests based on multiscale cross-entropy of BP and heart rate might integrate traditional HRV indices or baroreflex sensitivity assessments for stratifying the cardiovascular risk more efficiently or for better monitoring the progress of treatments or of cardiac rehabilitation protocols.

## Figures and Tables

**Table 1:** Sample entropy (*SampEn*) and systolic blood-pressure-pulse interval (SBP-PI) cross-*SampEn* by conditions and groups as mean (SD), with significance *p* of the factors *Group*, *Time*, and of their interaction.

					*p* Value	
		Day	Night	Group	Time	Time *Group
PI *SampEn*						
*m* = 1	NT	1.02 (0.21) *	1.31 (0.31)	0.25	<0.001	0.10
	HT	0.84 (0.14) **	1.32 (0.27)			
*m* = 2	NT	0.94 (0.23) *	1.22 (0.29)	0.31	<0.001	0.07
	HT	0.75 (0.15) **	1.27 (0.27)			
*m* = 3	NT	0.88 (0.23) *	1.06 (0.21)	0.50	<0.001	0.06
	HT	0.69 (0.17) **	1.14 (0.30)			
SBP *SampEn*						
*m* = 1	NT	1.29 (0.19)	1.41 (0.30)	0.45	<0.05	>0.99
	HT	1.37 (0.21)	1.45 (0.29)			
*m* = 2	NT	1.25 (0.19)	1.37 (0.29)	0.49	<0.05	0.92
	HT	1.30 (0.18)	1.42 (0.29)			
*m* = 3	NT	1.18 (0.18)	1.24 (0.25)	0.34	0.19	0.83
	HT	1.25 (0.18)	1.27 (0.28)			
DBP *SampEn*						
*m* = 1	NT	1.25 (0.24)	1.35 (0.32)	0.83	0.18	0.47
	HT	1.26 (0.26)	1.31 (0.29)			
*m* = 2	NT	1.20 (0.25)	1.30 (0.33)	0.83	0.14	0.68
	HT	1.19 (0.27)	1.26 (0.30)			
*m* = 3	NT	1.17 (0.25)	1.25 (0.32)	0.90	0.16	0.68
	HT	1.16 (0.27)	1.23 (0.31)			
SBP-PI cross-*SampEn*						
*m* = 1	NT	1.22 (0.13) *	1.47 (0.25)	0.78	<0.01	0.86
	HT	1.20 (0.15) *	1.43 (0.28)			
*m* = 2	NT	1.19 (0.15) *	1.46 (0.28)	0.70	<0.01	0.67
	HT	1.15 (0.14) **	1.42 (0.28)			
*m* = 3	NT	1.13 (0.15) *	1.33 (0.21)	0.83	<0.01	0.63
	HT	1.11 (0.15) **	1.30 (0.27)			

NT = normotensive group (8 subjects); HT = hypertensive group (8 subjects); *m* = embedding dimension; * and ** indicate significances *p*, respectively, <0.05 and <0.01 by *a posteriori* contrasts with False Discovery Rate correction according to [31].

**Table 2:** Averages of modified-multiscale entropy (*mMSE*) and SBP-PI modified multiscale cross entropy (*mXMSE*) over the ranges of scales corresponding to the HF, LF, VLF1, and VLF2 bands, by conditions and groups: mean (SD).

		HF		LF		VLF1		VLF2	
		Day	Night	Day	Night	Day	Night	Day	Night
PI *mMSE*									
*m* = 1	NT	1.31 (0.25)	1.17 (0.25)	1.34 (0.19)	1.19 (0.29)	1.27 (0.24)	0.93 (0.25)	1.18 (0.22)	0.90 (0.24)
	HT	1.05 (0.29)	1.19 (0.33)	1.12 (0.25)	1.21 (0.35)	1.08 (0.23)	1.01 (0.26)	1.03 (0.20)	0.96 (0.21)
*m* = 2	NT	1.23 (0.24)	1.06 (0.21)	1.26 (0.20)	1.06 (0.25)	1.17 (0.29)	0.75 (0.25)	1.08 (0.31)	0.77 (0.22)
	HT	0.97 (0.27)	1.10 (0.35)	1.04 (0.25)	1.06 (0.35)	0.99 (0.25)	0.81 (0.25)	0.92 (0.23)	0.81 (0.24)
*m* = 3	NT	1.14 (0.24)	0.96 (0.16)	1.18 (0.22)	0.92 (0.19)	1.11 (0.34)	0.64 (0.25)	1.00 (0.42)	0.70 (0.21)
	HT	0.89 (0.25)	1.02 (0.34)	0.97 (0.25)	0.94 (0.34)	0.92 (0.25)	0.67 (0.23)	0.88 (0.29)	0.70 (0.25)
SBP *mMSE*									
*m* = 1	NT	1.44 (0.19)	1.26 (0.28)	1.31 (0.18)	1.30 (0.26)	1.22 (0.18)	1.03 (0.24)	1.24 (0.24)	0.95 (0.26)
	HT	1.41 (0.15)	1.18 (0.36)	1.30 (0.15)	1.23 (0.37)	1.11 (0.18)	0.90 (0.29)	1.19 (0.22)	0.84 (0.25)
*m* = 2	NT	1.34 (0.21)	1.20 (0.28)	1.23 (0.20)	1.21 (0.25)	1.15 (0.19)	0.92 (0.24)	1.20 (0.33)	0.84 (0.25)
	HT	1.33 (0.17)	1.11 (0.36)	1.21 (0.17)	1.12 (0.36)	1.05 (0.19)	0.79 (0.27)	1.14 (0.26)	0.74 (0.26)
*m* = 3	NT	1.25 (0.19)	1.13 (0.27)	1.19 (0.22)	1.11 (0.23)	1.11 (0.23)	0.85 (0.24)	1.16 (0.45)	0.78 (0.26)
	HT	1.21 (0.15)	1.05 (0.35)	1.14 (0.19)	1.03 (0.35)	1.00 (0.20)	0.73 (0.27)	1.16 (0.31)	0.69 (0.27)
DBP *mMSE*									
*m* = 1	NT	1.45 (0.25)	1.34 (0.29)	1.28 (0.18)	1.33 (0.30)	1.08 (0.22)	1.01 (0.23)	1.08 (0.25)	0.95 (0.27)
	HT	1.32 (0.23)	1.25 (0.30)	1.26 (0.24)	1.24 (0.30)	1.08 (0.27)	0.92 (0.21)	1.06 (0.31)	0.84 (0.19)
*m* = 2	NT	1.38 (0.27)	1.28 (0.31)	1.20 (0.22)	1.22 (0.32)	1.00 (0.26)	0.86 (0.27)	1.02 (0.29)	0.82 (0.28)
	HT	1.27 (0.25)	1.20 (0.31)	1.18 (0.27)	1.14 (0.30)	0.99 (0.28)	0.79 (0.20)	0.99 (0.31)	0.73 (0.20)
*m* = 3	NT	1.32 (0.29)	1.21 (0.31)	1.16 (0.26)	1.11 (0.32)	0.96 (0.30)	0.77 (0.30)	0.96 (0.33)	0.74 (0.29)
	HT	1.19 (0.24)	1.14 (0.31)	1.10 (0.27)	1.05 (0.28)	0.93 (0.28)	0.71 (0.19)	0.97 (0.29)	0.66 (0.18)
SBP-PI *mXMSE*									
*m* = 1	NT	1.41 (0.20)	1.33 (0.21)	1.39 (0.12)	1.38 (0.18)	1.34 (0.14)	1.13 (0.16)	1.33 (0.14)	1.08 (0.19)
	HT	1.32 (0.16)	1.26 (0.29)	1.31 (0.10)	1.33 (0.30)	1.20 (0.11)	1.11 (0.24)	1.25 (0.16)	1.06 (0.26)
*m* = 2	NT	1.33 (0.20)	1.25 (0.21)	1.32 (0.13)	1.26 (0.16)	1.28 (0.15)	0.98 (0.18)	1.25 (0.20)	0.99 (0.21)
	HT	1.24 (0.18)	1.18 (0.29)	1.22 (0.11)	1.21 (0.28)	1.12 (0.14)	0.96 (0.23)	1.16 (0.24)	0.97 (0.31)
*m* = 3	NT	1.23 (0.18)	1.17 (0.18)	1.26 (0.14)	1.14 (0.14)	1.21 (0.15)	0.89 (0.19)	1.20 (0.28)	0.94 (0.22)
	HT	1.14 (0.16)	1.10 (0.29)	1.14 (0.12)	1.10 (0.26)	1.06 (0.16)	0.84 (0.20)	1.11 (0.30)	0.95 (0.34)
